# Koch–Haaf reaction of adamantanols in an acid-tolerant hastelloy-made microreactor

**DOI:** 10.3762/bjoc.7.149

**Published:** 2011-09-15

**Authors:** Takahide Fukuyama, Yu Mukai, Ilhyong Ryu

**Affiliations:** 1 Department of Chemistry, Graduate School of Science, Osaka Prefecture University, Sakai, Osaka 599-8531, Japan

**Keywords:** continuous flow system, hastelloy, Koch–Haaf reaction, microreactor

## Abstract

The Koch–Haaf reaction of adamantanols was successfully carried out in a microflow system at room temperature. By combining an acid-tolerant hastelloy-made micromixer, a PTFE tube, and a hastelloy-made microextraction unit, a packaged reaction-to-workup system was developed. By means of the present system, the multigram scale synthesis of 1-adamantanecarboxylic acid was achieved in ca. one hour operation.

## Introduction

The recent evolution of microreactor technology has allowed synthetic chemists to use this precisely sophisticated reaction apparatus in place of the well-established glassware batch flask [1–10]. Microreactors are expected to have a significant impact on chemical synthesis and production because of their many advantageous characteristics, such as highly efficient mixing, efficient heat transfer ability, precise control over the residence time, and high operational safety. We have studied and developed practical organic syntheses using flow microreactors, and we have reported thus far examples of Pd-catalyzed coupling reactions [11–13], radical reactions [14–16], and photoreactions [17–21].

Carbonylation reactions are a powerful tool for the introduction of carbon monoxide into organic molecules, and we also reported that Pd-catalyzed carbonylation [13] and radical carbonylation [16] could be successfully carried out in a continuous microflow system with higher efficiency than in a batch autoclave system. In this study, we focused on the carbonylation of carbocation intermediates carried out in a continuous microflow system [22–24]. The Koch–Haaf reaction [25], that is the carbonylation of alcohols or olefins with formic acid in the presence of a strong acid, is an important reaction for the preparation of carboxylic acids, which are widely used in organic synthesis [26–31]. Since the Koch–Haaf reaction is highly exothermic, the reaction is typically carried out at controlled temperature by means of a cooling bath, such as an ice bath, and with carefully controlled slow addition of reagents through an addition funnel. The temperature control causes a serious problem especially for large scale synthesis. Herein, we report that the Koch–Haaf reaction in a microflow reactor can be carried out at room temperature without any cooling equipment. The employed hastelloy-made microreactor system was compatible with corrosive (strongly acidic) conditions and confirmed for gram scale (7.1 g) synthesis of 1-adamantanecarboxylic acid in ca. 1 h operation.

## Results and Discussion

**Figure 1:**
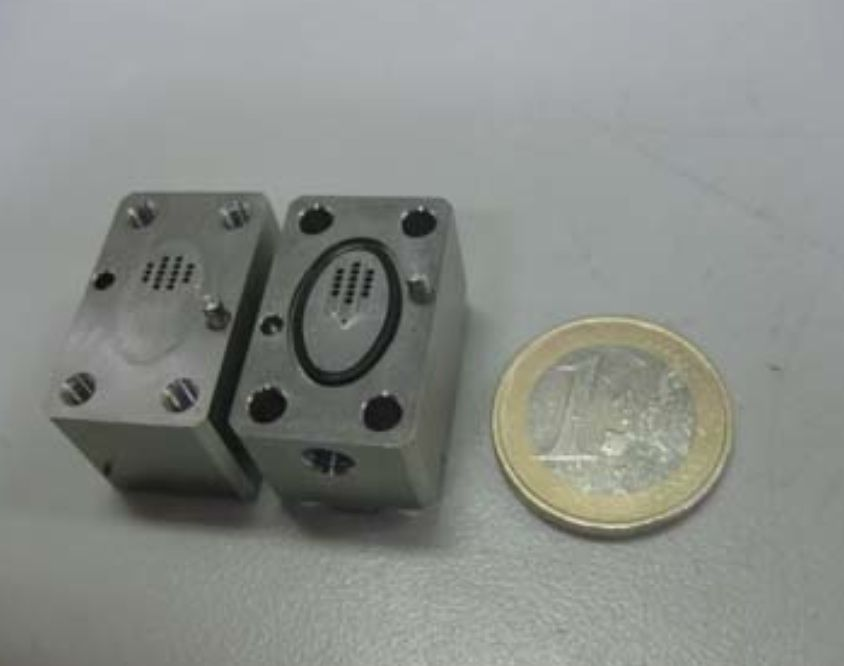
Hastelloy-made micromixer (MiChS β-150H).

**Figure 2:**
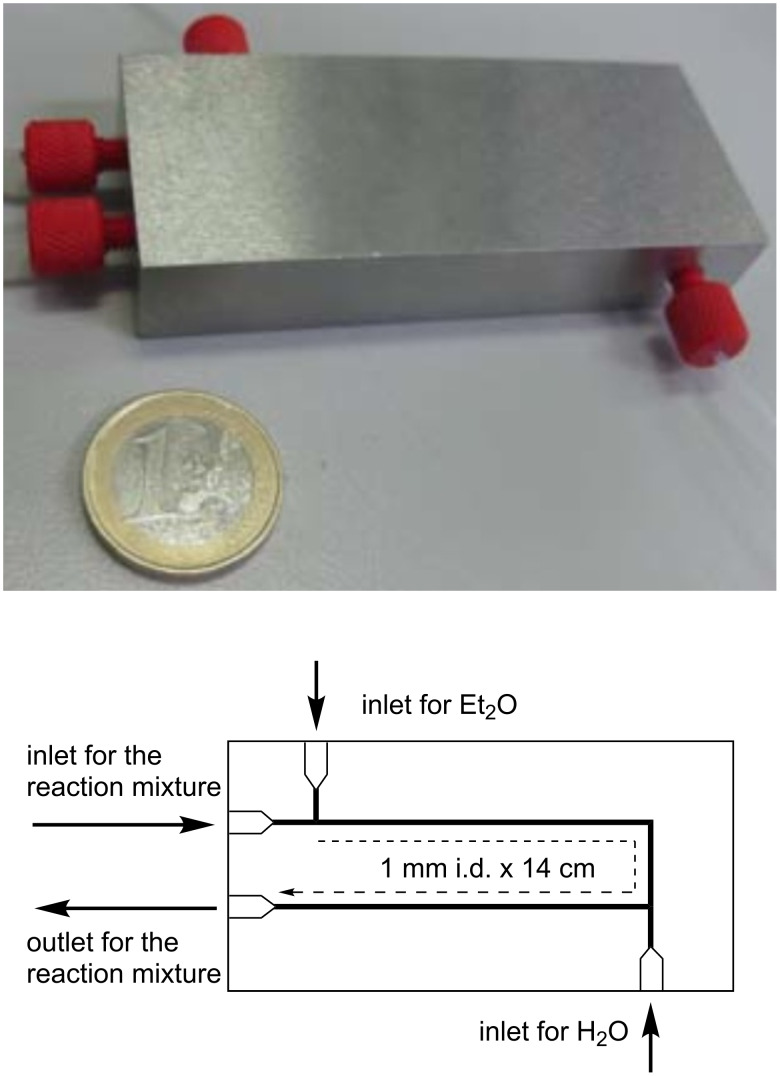
Hastelloy-made microextraction unit.

**Figure 3:**
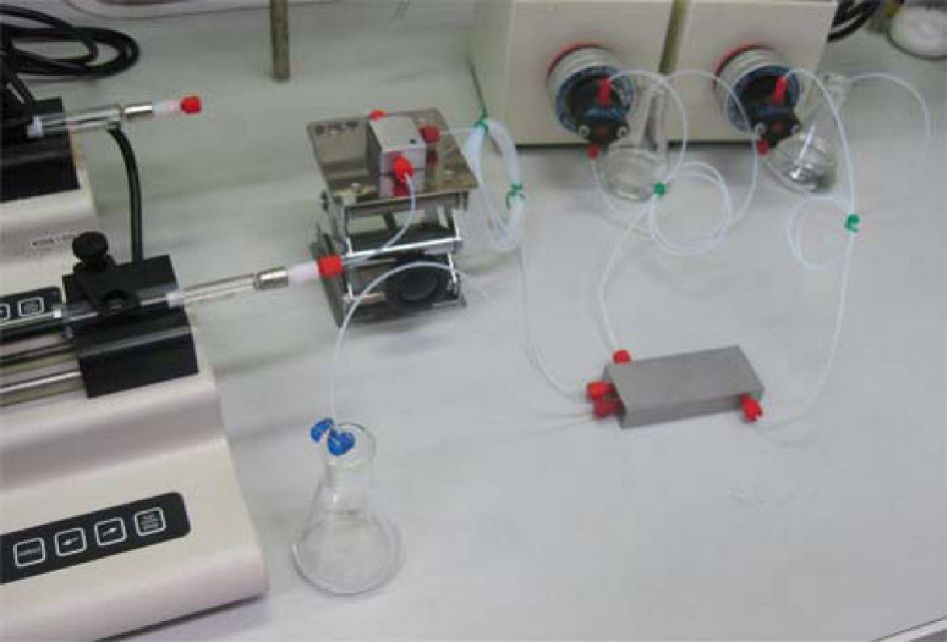
Acid-tolerant microflow system used for the Koch–Haaf reaction.

The carbonylation reaction of 1-adamantanol (**1a**) was investigated in a microflow system as a model reaction. Since the Koch–Haaf reaction requires the use of concentrated sulfuric acid, an acid-tolerant system is essential. For this study, we employed a combination of a hastelloy-made micromixer (MiChS, β-150H) having 150 μm reactant inlet holes and 200 μm × 300 μm channels (Figure 1), and a PTFE tube (1.0 mm i.d. × 3 m, inner volume: 2.36 mL) as a residence time unit. To this reactor system, a hastelloy-made microextraction unit (a flow-workup system) was attached (Figure 2 and Figure 3). The microextraction unit has three inlets and one outlet (channel size: 1 mm i.d. × 14 cm). The reaction mixture was mixed at T-shaped junctions with Et_2_O and water, and a biphasic mixture was collected from the outlet.

**Scheme 1:**
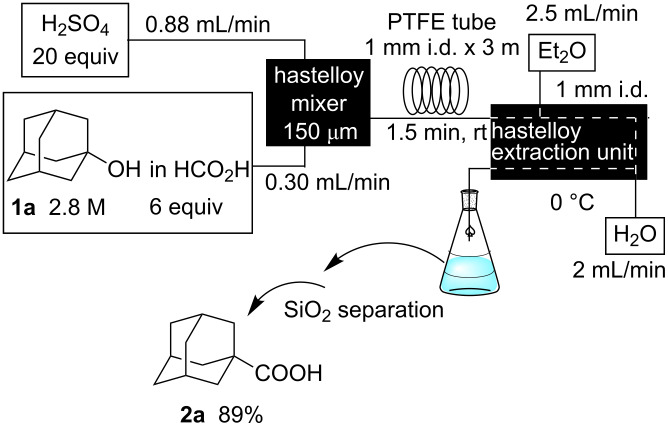
Synthesis of 1-adamantanecarboxylic acid (**2a**) in a microflow system.

1-Adamantanol (**1a**) dissolved in HCOOH (flow rate: 0.30 mL/min) and 98% H_2_SO_4_ (flow rate: 0.88 mL/min) were mixed in the micromixer at room temperature, and the resulting reaction mixture was fed into the PTFE tube and then into the extraction unit, in which Et_2_O (flow rate: 2.5 mL/min) and water (2 mL/min) were introduced to extract the carbonylation product and remove excess acids (Scheme 1). The biphasic mixture was collected in a flask and the ether layer was concentrated in vacuo. 1-Adamantanecarboxylic acid (**2a**) was obtained in 89% isolated yield after purification by silica gel column chromatography. While the residence time was a priori expected to be 2 min based on the total flow rate of the reagents and inner volume of the residence time unit, the observed residence time was 1.5 min due to a plug flow by the CO gas generated.

For comparison, we also carried out the batch reaction in a 50 mL glass flask on 4 mmol scale to give **2a** in 92% yield. In the batch reaction, the careful addition of a solution of **1a** in formic acid over a period of 5 min and cooling in an ice bath were necessary to achieve good results. Indeed, without a cooling bath, we observed that the temperature of the reaction mixture rose up to 50–60 °C. It is therefore remarkable that the reaction in the microflow system can be performed successfully at room temperature without any cooling unit.

**Scheme 2:**
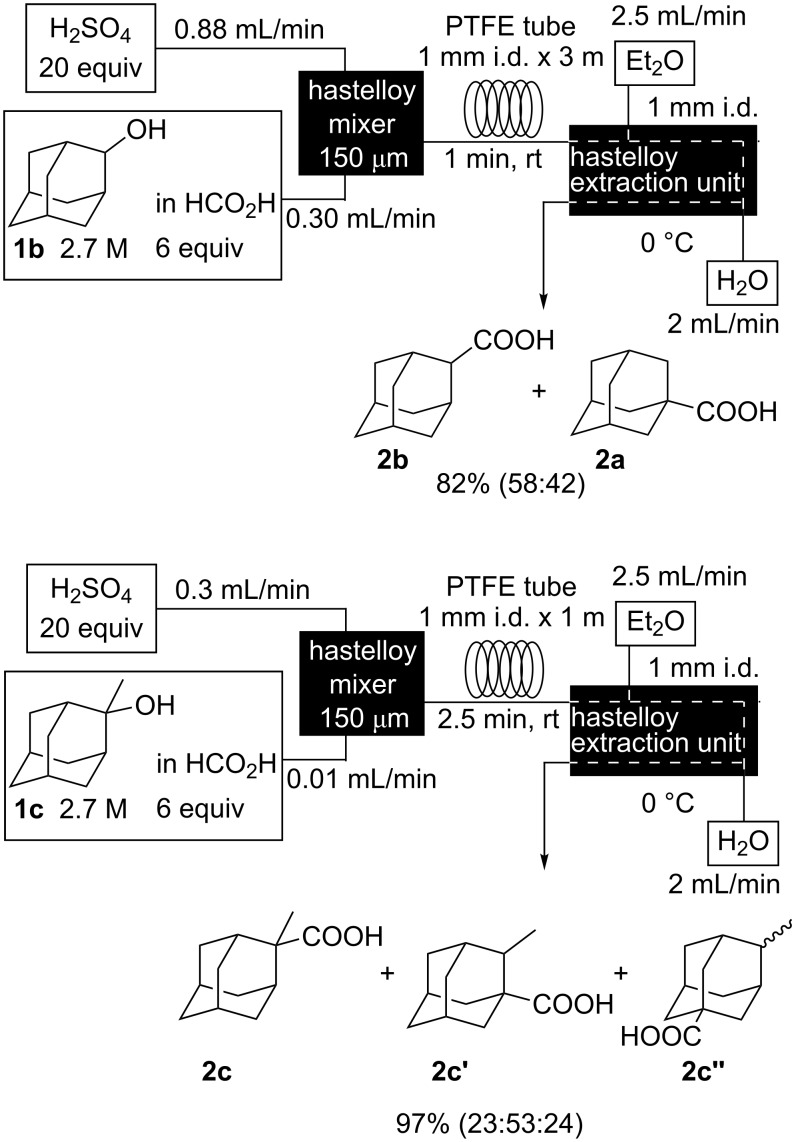
Koch–Haaf reaction of **1b** and **1c** in a microflow system.

We then investigated the reaction of some other adamantanols, such as that of 2-adamantanol (**1b**) and 2-methyl-2-adamantanol (**1c**) (Scheme 2). The reaction of **1b** in a microflow system gave a mixture of 2-adamantanecarboxylic acid (**2b**) and 1-adamantanecarboxylic acid (**2a**) (82% total yield, **2b**:**2a** = 58:42), in which the latter compound originated from the isomerized tertiary cation, which derived from the initially formed secondary cation. The batch reaction gave a mixture of **2b** and **2a** in 65% total yield with a greater proportion of the rearranged product (**2b**:**2a** = 14:86). The reaction of 2-methyl-2-adamantanol (**1c**) resulted in a mixture of the carboxylated products, **2c**, **2c'**, and **2c''** in 97% total yield (**2c**:**2c'**:**2c''** = 23:53:24). The batch reaction resulted in an inferior yield with more of the rearranged products (83% yield, **2c**:**2c'**:**2c''** = 19:62:19). All results are summarized in Table 1.

**Table 1:** Koch–Haaf reactions of adamantanols.^a^

Entry	**1**	Reactor	Conditions	Product (yield)^b^

1	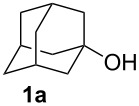	microflow	*T*: 20 °C flow rate (**1a**/HCO_2_H): 0.30 mL/min flow rate (H_2_SO_4_): 0.88 mL/min residence time: 2 min^c^ residence time: 1.5 min^d^	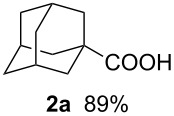
2	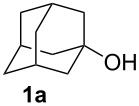	batch	*T*: 15–20 °C addition time: 5 min reaction time: 2 min	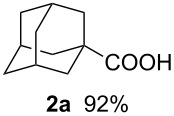
3	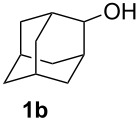	microflow	*T*: 20 °C flow rate (**1b**/HCO_2_H): 0.30 mL/min flow rate (H_2_SO_4_): 0.88 mL/min residence time: 2 min^c^ residence time: 1 min^d^	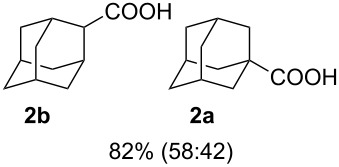
4	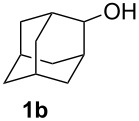	batch	*T*: 17–20 °C addition time 5 min reaction time 1 min	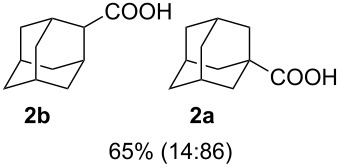
5	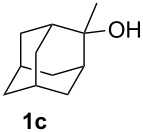	microflow	*T*: 20 °C flow rate (**1c**/HCO_2_H): 0.01 mL/min flow rate (H_2_SO_4_): 0.3 mL/min residence time: 20 min^c^ residence time: 2.5 min^d^	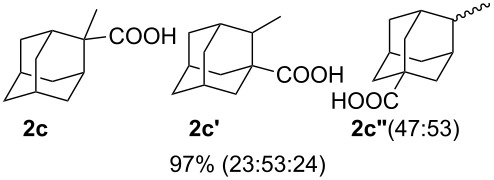
6	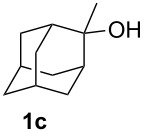	batch	*T*: 17–20 °C addition time: 3 min reaction time: 10 min	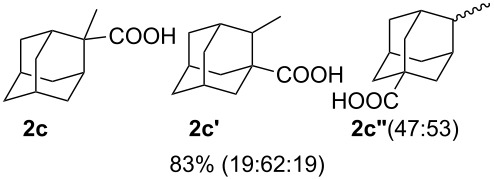

^a^**1** (4 mmol), HCOOH (6 equiv), H_2_SO_4_ (20 equiv); ^b^isolated yield after column chromatography on SiO_2_; ^c^calculated; ^d^observed.

**Scheme 3:**
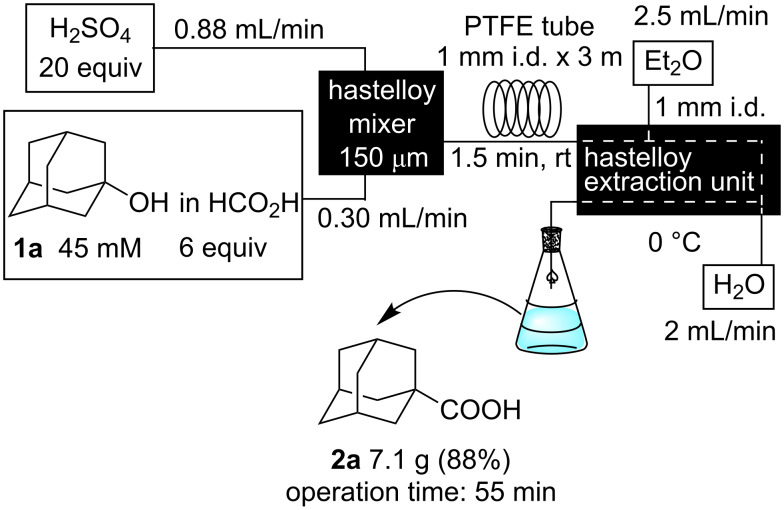
Multigram scale flow synthesis of 1-adamantanecarboxylic acid (**2a**).

Multigram scale synthesis of **2a** from **1a** was carried out in a continuous flow reaction. When the reaction of **1a** (45 mmol) was performed for 55 min, 7.1 g of **2a** was obtained in 88% yield, demonstrating that the present microflow system can be used for multigram scale synthesis without any problems (Scheme 3).

## Conclusion

In this work, we demonstrated that the Koch–Haaf reaction of adamantanols was successfully carried out in an acid-tolerant microflow system comprising a hastelloy-made micromixer, a PTFE tube, and a hastelloy-made microextraction unit. Unlike in the batch system, the reaction could be carried out at room temperature without any cooling equipment. The employed reaction-to-workup system was useful for the multigram scale synthesis of 1-adamantanecarboxylic acid (**2a**). We are now expanding the system to other cationic systems and the results will be published in due course.

## Experimental

**Typical procedure for Koch–Haaf reaction in a microflow system. Multigram scale synthesis of 1-adamantanecarboxylic acid (2a).** 1-Adamantanol (**1a**, 60 mmol, 9.2 g) was dissolved in 96% HCOOH (360 mmol, 16.6 g), and the solution was placed in a 50 mL syringe (22.3 mL), which was then attached to a syringe pump. Concentrated H_2_SO_4_ (99%) (1.2 mol, 64 mL) was placed in 100 mL syringe. These liquids were mixed in the hastelloy micromixer (150 μm) (flow rate: **1a** in HCOOH = 0.3 mL/min, H_2_SO_4_ = 0.88 mL/min). The resulting reaction mixture was then fed into the residence time unit (PTFE tube, 1 mm i.d. × 3 m). The residence time was observed to be 1.5 min. The mixture of products was fed into the hastelloy-made extraction unit, which was cooled by an ice/water bath. Et_2_O (2.5 mL/min) and water (2 mL/min) were fed into the extraction unit. The mixture that was eluted during the first 5 min was discarded and the portion that followed was collected for 55 min (**1a**: 45 mmol). The ethereal layer was separated, and washed with 1.4 N KOH aq. The aqueous layer was acidified with 1 N HCl and extracted with Et_2_O. The organic layer was dried over MgSO_4_, filtered, and evaporated. 1-Adamantanecarboxylic acid (**2a**) was obtained in 88% yield as a white solid (7.1 g, mp 171–172 °C). The obtained product was identified by comparison of the ^1^H NMR and ^13^C NMR spectra with those of commercially available authentic samples. All other products, **2b**, **2c**, **2c'**, and **2c''** were identified by means of NMR spectroscopy by comparison with literature data [32–33].

### Typical procedure for Koch–Haaf reaction in a batch reaction system

In a 50 mL two-necked round bottom flask, 99% H_2_SO_4_ (80 mmol, 7.85 g) was placed. A solution of 1-adamantanol (**1a**, 4 mmol, 613 mg) in 96% HCOOH (24 mmol, 1.01 g) was added through a dropping funnel over a period of 5 min, while the temperature of the reaction mixture was maintained at 15–20 °C in an ice/water bath. The reaction mixture was stirred at 15–20 °C for an additional 2 min, poured into ice/water and extracted with Et_2_O. The ethereal layer was washed with 1.4 N KOH aq, and the aqueous layer was acidified with 1 N HCl and extracted with Et_2_O. The organic layer was dried over MgSO_4_, evaporated and purified by column chromatography on SiO_2_. Compound **2a** was obtained in 92% yield (667 mg). The reaction of **1b** and **1c** was carried out by a similar procedure.
